# Socioeconomic inequalities in insulin initiation among individuals with type 2 diabetes – A quasi-experimental nationwide register study

**DOI:** 10.1016/j.ssmph.2022.101178

**Published:** 2022-08-09

**Authors:** Hanna Rättö

**Affiliations:** The Social Insurance Institution, Kela Research, Helsinki, PO Box 450, 00056, Kela, Finland

**Keywords:** Diabetes, Disparity, Drug co-payment, Health reform, Inequality, Inequity

## Abstract

**Background:**

Inequalities in access to care can translate to or strengthen existing inequalities in health if people of lower socioeconomic positions do not have equal access to care. I study insulin initiation among individuals with type 2 diabetes and examine whether a reform increasing the co-payment of non-insulin antidiabetics in Finland in 2017 had an inequitable effect on the initiation. In the treatment of type 2 diabetes, insulin is recommended only in later stages and remains covered by the National Health Insurance at a rate of 100%.

**Data and methods:**

I evaluated the effect of the reform with Cox proportional hazard modelling using nationwide person-level register data from 2011 to 2019. Exploiting a quasi-experimental design rising from the introduction of the reform allows for consideration of causality.

**Results:**

I found that the risk of insulin initiation was lower in the later years of the study period. Additionally, individuals in lower socioeconomic positions had a higher risk of initiation. However, I did not find inequalities in how the reform affected the risk of insulin initiation between income quintiles.

**Conclusions:**

Co-payments are unlikely to be the most influential factor behind persisting inequalities in insulin initiation among individuals with type 2 diabetes in Finland. Lower risk in the later years aligns with developing treatment practices of type 2 diabetes.

## Highlights

• Individuals with type 2 diabetes in lower socioeconomic positions had a higher risk of insulin initiation.
• The risk of insulin initiation was lower in the later years of the study period (2011-2019) in all income quintiles.
• No inequalities in the impact of the co-payment increase in non-insulin antidiabetics in insulin initiation were found.

## 1 Introduction

Affordable medicines are acknowledged as a key requirement for functional health care (European Commission, 2020). Health systems protect population health by providing access to necessary care with affordable cost. A well-functioning health care system can also help to mediate the consequences of determinants of ill health between people of different socioeconomic positions. (WHO, 2008.)

Several studies have indicated that even when needs are accounted for, health care use is biased towards those with higher income also in affluent countries (Blomgren & Virta, 2020; van Doorslaer et al., 2000, 2006). Even in universal health systems, coverage also depends on the range of covered services and the degree of cost sharing. Coverage for pharmaceuticals is often less comprehensive than coverage for inpatient and outpatient care (OECD, 2021.). Economic access, i.e. affordability, is one of the key dimensions of access to health care (Penchansky & Thomas, 1981) that, for example, the World Health Organization (WHO, 2005) mentions when describing prerequisites of equitable access to health care.

In recent years, austerity measures aiming to curb the increasing health budgets have emerged in several high-income countries (Arcà, Principe, & Van Doorslaer, 2020; Stadhouders, Kruse, Tanke, Koolman, & Jeurissen, 2019). In addition to cost savings, unintended outcomes have also been reported (Arcà et al., 2020; Lavikainen, Aarnio, Jalkanen, et al., 2020). As part of austerity measures targeted at the pharmaceutical budget in Finland, a reform increasing the co-payments of non-insulin antidiabetic medicines was introduced in 2017 (hereinafter: the reform). The reform raised concerns about increased use of insulin in the treatment of type 2 diabetes for economic rather than clinical reasons because the co-payment of insulin was not affected by the reform and remained fully covered (with a fixed fee of €4.50 per dispensing) (Government Proposal 184/2016; Lahtela, 2017).

Evolving treatment practices and the extending repertoire of antihyperglycemic agents in the market for type 2 diabetes have pushed insulin to even later lines of treatment because newer non-insulin antidiabetics are likely initiated before insulin (Järvinen, Laine, & Eriksson, 2016; Niskanen & Laine, 2020). Previous studies have suggested that insulin initiation in individuals with type 2 diabetes is associated with age and the existence of other illnesses (Abu-Ashour, Chibrikova, Midodzi, Twells, & Gamble, 2017; Ojala et al., 2021; Reach, Le Pautremat, & Gupta, 2013; Spoelstra et al., 2002). Early insulin initiation has also been associated with lower socioeconomic position, measured as household income and education or employment status (Abu-Ashour et al., 2017; Reach et al., 2013).

Increased cost sharing has previously been found to reduce the utilization of even necessary medicines (Kiil & Houlberg, 2014; Luiza et al., 2015; Sinnott, Buckley, O'Riordan, Bradley, & Whelton, 2013). Patients’ sensitivity to medicine co-payments and the potentially inequitable impact of said co-payment has been recognized also in Finland (Hamina, Tanskanen, Tiihonen, & Taipale, 2020; Tervola, Aaltonen, & Tallgren, 2021). Cost sharing can also affect the choice of medicine if treatments with different costs are available (Saito, Davis, Harrigan, Juarez, & Mau, 2010). Potentially, this is a problem; if the therapeutic values of the switched treatments are not equal, patients might be left with suboptimal treatment due to financial reasons.

### 1.1 Aims of the study

Based on previous literature, I formulated a three-part hypothesis to study factors associated with insulin initiation (hypotheses 1 and 2) and the effect of the reform (hypothesis 3).

Hypothesis 1 (H1) The risk of insulin initiation is lower in the later years of the study period.

Hypothesis 2 (H2) The risk of insulin initiation is lower in individuals of higher socioeconomic positions.

Hypothesis 3 (H3) The reform affected the risk of insulin initiation in individuals of lower socioeconomic positions more harmfully than it did those of higher socioeconomic positions.

To study the hypotheses, I used comprehensive Finnish register data from 2011 to 2019 and the quasi-experimental setting rising from the introduction of the reform. Using Cox proportional hazard models, I first investigated factors associated with the risk of insulin initiation and then compared the change in risk in individuals with different income levels. When investigating causal inference, individuals purchasing non-insulin antidiabetic medicines in 2014 were used as a control group unaffected by the reform.

The study produces timely causal evidence on health-related outcomes of austerity measures targeted at the health sector. The study also adds to the conversation on the mechanisms that enable the persistence of socioeconomic health differences.

## 2 Materials and methods

In Finland, all permanent residents are entitled to reimbursements for outpatient prescription medicines classified as reimbursable under the National Health Insurance (NHI) (Health Insurance Act 1224/2004). A universal basic reimbursement rate applies to all reimbursable medicines (40% of the retail price in 2019). In addition, there are two disease-based special reimbursement categories for severe and chronic diseases: higher (covering 100% of the retail price with a fixed co-payment of €4.50/purchase in 2019) and lower (covering 65% of the retail price in 2019). Entitlement to special reimbursement can be granted to patients based on a doctor's certificate (Kela, 2019.). An annual co-payment ceiling (€572 in 2019) for reimbursed medicines applies, after which patients pay a fixed fee (€2.50 per product per dispensing in 2019) for the rest of the calendar year. Since 2016, medicine reimbursements have applied only after patients pay an initial annual deductible of €50 out-of-pocket (children and youth aged 18 or under are exempt).

Prior to 2017, the special reimbursement rate for antidiabetic medicines was 100%. After the reform, special reimbursement was 65% for non-insulin antidiabetic medicines and 100% for insulins. Based on prospective microsimulations, almost 30% of patients with type 2 diabetes were expected to face an increase of €100 or more in their annual co-payment expenditure, the average increase being over €70 per patient (Government Proposal 184/2016; Kurko, Heino, Martikainen, & Aaltonen, 2018).

### 2.1 Data

This study is based on individual-level nationwide register data on all antidiabetic medicine purchases (i.e. insulins belonging to Anatomical Therapeutic Chemical (ATC) (WHOCC, 2015) class A10A and non-insulin antidiabetics belonging to class A10B) reimbursable by the NHI in Finland between 2011 and 2019. The purchases are recorded in the Dispensations reimbursable under the NHI scheme register maintained by the Social Insurance Institution (Kela). From the register, I collected information on the ATC class of the medicine, the date of purchase, and the age, sex, and pseudonymized ID of the individual. By using pseudonymized ID, disease-based special reimbursement entitlements (morbidity), possible date of death, and taxable income were linked from respective registers (See also Supplemental Table 1.).

### 2.2 Study sample and research design

To examine whether the impact of out-of-pocket payment increase varied with patient's income level, I defined two groups. Treatment group consisted of all insulin-naïve (no insulin purchases during the preceding three years) individuals who purchased non-insulin antidiabetic medicines in 2017 and 2016, and the control group consisted of all insulin-naïve individuals who purchased non-insulin antidiabetic medicines in 2014 and 2013.

Both groups were then followed for a maximum of three years (2017–2019 and 2014–2016 for treatment and control groups, respectively) to inspect the insulin initiation. From the definition of the treatment and control groups follows that a number of patients belong to both groups. However, because of the definitions of the groups, a patient can only initiate insulin once. The patients’ characteristics are also defined separately for each group at the beginning of the follow-up.

### 2.3 Outcome variable: insulin initiation

Individuals in treatment and control groups who purchased insulin during the follow-up were defined as initiating insulin treatment, and the date of the individuals’ first insulin purchase during the follow-up period was defined as the date of insulin initiation. Time to insulin initiation is the time between the beginning of the follow-up (January 1, 2017/2014 for treatment and control groups, respectively) and the date of the first insulin purchase.

### 2.4 Controls and characteristics

Individuals were grouped into income quintiles based on their personal taxable income at the beginning of the follow-up. Quintile limits were calculated based on taxable income of the individuals in the treatment group, and consumer price index (Statistics Finland, 2022a) was used to harmonize the price level. The first quintile includes individuals with income in the lowest 20%, and the fifth quintile consists of individuals with income in the highest 20%. The quintile limits do not necessarily reflect those of the entire population but rather the part of the population targeted by the reform.

To account for other existing illnesses, individuals who were granted at least one disease-based entitlement to reimbursement (other than diabetes) were defined as having a comorbidity. In addition, the age and sex of the patient were used as controls. All individual-level information (including income) is accounted for from the end of the year before the follow-up, i.e. 2016 for treatment group and 2013 for control group.

To assess the similarities between treatment and control groups further, the time since diabetes reimbursement entitlement was granted, the share of individuals purchasing newer non-insulin antidiabetic medicines, the share of individuals purchasing insulin, and the share of patients dying are also defined for both groups. The characteristics of individuals in treatment and control groups are presented in Table 1.

**Table 1:** Characteristics of individuals in treatment and control groups at the beginning of the follow-up.

	Treatment	Control
Number of individuals	226,614	207,749
Share of females (%)	46	47
Mean age (years)	67	66
Mean taxable income (EUR, in 2016 prices)	27,521	26,561
Mean number of granted disease-based special reimbursement entitlements	1.8	1.8
Share with a comorbidity, i.e. granted disease-based special reimbursement entitlements excluding diabetes (%)	54	54
Share with diabetes-related special reimbursement entitlement (%)	80	75
Duration of diabetes at the beginning of follow-up, i.e. time since diabetes-related special reimbursement entitlement was granted (years)	5.1	4.2
Share of patients purchasing insulin during follow-up (%)	7.8	8.4
Share of patients purchasing newer non-insulin antidiabetics* during follow-up (%)	23	10
Share of patients dying during follow-up (%)	7	7

* GLP-1 analogues (A10BJ) and SGLT2 inhibitors (A10BK).

The characteristics of the individuals in compared groups are quite similar. In both groups, a little less than half of the individuals are female. The share of females is slightly higher in control group, whereas the mean taxable income is slightly higher in treatment group. On average, individuals in both groups are over 65 years old, and 7% of the individuals die during the three-year follow-up in both groups. The mean age is, however, slightly higher in treatment group. As treatment group was drawn from a later time point than control group (2017 vs. 2014), the rising average age of Finnish population (Statistics Finland, 2022b) is likely reflected in the higher mean age.

On average, individuals in both groups have 1.8 disease-based special reimbursement entitlements, including the possible entitlement for diabetes. A little more than half of the individuals in both groups have a comorbidity. The share of individuals with diabetes-related special reimbursement entitlement is slightly higher in treatment group (80% vs. 75%), as is the time since diabetes reimbursement entitlement was granted (5.1 vs. 4.2 years). It is likely that this, at least partly, reflects the changes in the criteria for diabetes-related reimbursement entitlement. Prior to 2011, patients with type 2 diabetes were eligible for the special reimbursement only after using an antidiabetic medicine for six months. The use of newer non-insulin antidiabetic medicines (GLP-1 analogues, SGLT2 inhibitors) is more prevalent in the treatment group (23% vs. 10%) because of the gradually increasing uptake of such medicines during the study period (see also Supplemental Table 2). Conversely, the prevalence of insulin use is lower (7.8% vs. 8.4%).

### 2.5 Statistical methods

**Fig. 1:**
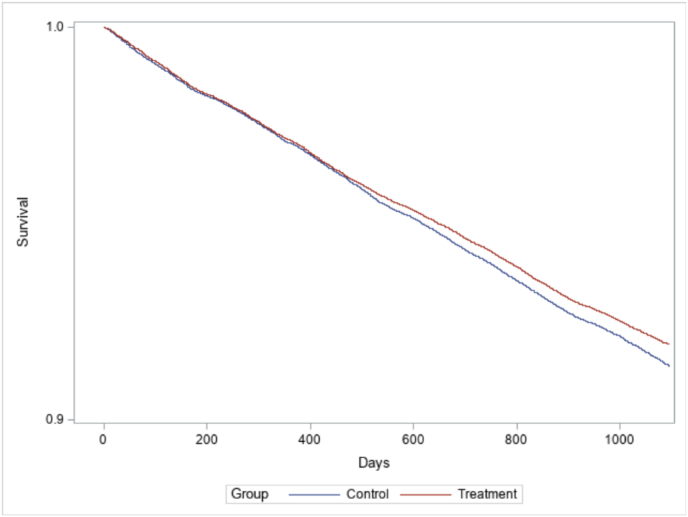
Kaplan-Meier curves for insulin initiation in treatment and control groups. (Y-axis from 0.9 to 1 for clarity.)

I first assessed insulin initiation with descriptive Kaplan-Meier curves and then with Cox proportional hazard models. Proportional hazard models assume that the hazard ratios of studied outcomes between compared groups are constant throughout the analyzed period (Zwiener, Blettner, & Hommel, 2011). The assumption was assessed based on Kaplan-Meier curves and, as hazard rates of compared groups were found to be parallel, deemed to hold (Fig. 1).

Cox proportional hazard models evaluate the share of followed-up units ‘at risk’ of the event of interest at any given time point and allow to inspect the impact of several factors simultaneously (Zwiener et al., 2011). Individuals in the analysis were followed for a maximum of three years to define the risk of insulin initiation in compared groups. In the analysis, individuals who did not purchase insulins during the maximum follow-up were marked censored. The data also contained information on the (possible) time of death of the individual, and, thus, it was possible to distinguish the individuals who ceased to be ‘at risk’ for insulin initiation because of their death. As both groups had a mean age of over 65, the sub-hazard of insulin initiation was estimated with death as a competing risk (e.g. Austin, Lee, & Fine, 2016). Results are presented as hazard ratios (HR) with their 95% Wald confidence limits (CL), and, in addition, Wald's test is used to test the statistical significance of included terms. All data management and statistical analysis were done using SAS version 9.4.

## 3 Results

Table 2 presents the results for Cox proportional hazard modelling for insulin initiation estimated with death as a competing risk. Model 1 includes the effects of the group (treatment/control) and income quintile on insulin initiation in individuals with type 2 diabetes. To inspect whether the impact of the reform on insulin initiation differed between income levels, an interaction term between group and income quintile was included in model 2. Both models also take into account the age and sex of the individual as well as the (possible) existence of a comorbidity.

**Table 2:** Results for Cox proportional hazard modelling estimated with death as a competing risk.

	Model 1		Model 2	
	HR	95% CL	HR	95% CL
*Group (ref: control, at V in model 2)*				
Treatment	0.935	0.915–0.954	0.915	0.870–0.963
*Income quintile (ref: V, at control in model 2)*				
I vs V	1.417	1.369–1.467	1.391	1.325–1.460
II vs V	1.253	1.209–1.298	1.244	1.184–1.307
III vs V	1.133	1.093–1.173	1.127	1.072–1.184
IV vs V	1.072	1.035–1.111	1.044	0.997–1.103
Male (ref: female)	1.152	1.127–1.177	1.152	1.127–1.177
Comorbidity (ref: no comorbidity)	1.560	1.526–1.594	1.560	1.526–1.594
Age (years)	1.002	1.001–1.003	1.002	1.001–1.003
Group*Income quintile -interaction			NS*	

*Wald's test, p = 0.6780.

Results from model 1 suggest that the risk of insulin initiation was lower in treatment group than in control group (HR 0.94, 95% CL 0.92–0.96). Thus, in general, the risk of initiating insulin was lower after the reform than before. This supports hypothesis 1. Results also imply that, compared to the highest quintile, the risk of insulin initiation is higher in the lower quintiles. The finding aligns with hypotheses 2. Being male, higher age and the existence of a co-morbidity are also associated with higher risk.

Finally, as the interaction between group and income quintile is not significant (p = 0.6780), the results from model 2 indicate that the effect of income level on insulin initiation is similar in treatment and control groups. Contrary to hypothesis 3, the effect of the reform did not differ by income level, and individuals with lower income were not more harmfully affected by the reform.

## 4 Discussion

Socioeconomic inequalities among diabetes patients have been demonstrated in previous research (e.g. Hill-Briggs et al., 2020). In this paper, I studied the risk of insulin initiation in individuals with type 2 diabetes in Finland. In addition, I examined whether the reform increasing out-of-pocket payments of non-insulin antidiabetics resulted in widening inequalities in said risk. Based on previous literature, I formulated a three-part hypothesis. First, I hypothesized that, in general, the risk of insulin initiation would be lower in the later years of the study period (H1) and in individuals of higher socioeconomic positions (H2). Additionally, I hypothesized that the reform would affect the risk of insulin initiation in individuals of lower socioeconomic positions more harmfully than those of higher socioeconomic positions (H3). Findings from the study supported the first two hypotheses (H1, H2). However, contrary to the third hypothesis (H3), the results showed that the change in risk after the reform did not significantly differ between income quintiles.

The lower risk of insulin initiation after the reform most likely reflects the changing treatment practices of type 2 diabetes and newer non-insulin antidiabetic medicines entering clinical practice also in Finland (Järvinen et al., 2016; Niskanen & Laine, 2020). In type 2 diabetes, initiation of newer antidiabetics is, in most cases, recommended before insulin. This, in turn, likely reflects as a decreasing trend in insulin initiation and specifically a lower risk after the reform, as suggested in hypothesis 1. The increasing use of newer antidiabetics in treatment group offers some additional support for hypothesis 1.

In line with hypothesis 2, the risk of insulin initiation was found to be lower in higher income quintiles already before the reform and remaining so after. There are likely several reasons for these prevailing socioeconomic inequalities. In Finnish Current Care Guidelines, insulin is typically recommended in Type 2 diabetes (2018) e.g. to control difficult hyperglycemia or if patients show signs of insulin deficiency. These conditions are often associated with obesity (Kahn, Hull, & Utzschneider, 2006). As lower income is associated with several lifestyle-related risk factors, such as smoking or habits related to eating and physical activity, prevailing risk differences between income quintiles probably reflect, at least partly, the general socioeconomic stratification in health. A regional study in Finland did not find income to be a factor associated with the risk of insulin initiation (Ojala et al., 2021). However, the information on income was based on area-based measure, complicating comparison with the current study. The effects for other factors included in the estimations (age, sex, existence of a co-morbidity) largely align with previous studies (Abu-Ashour et al., 2017; Ojala et al., 2021; Reach et al., 2013; Spoelstra et al., 2002) and the disease prognosis.

Contrary to hypothesis 3, inequities in insulin initiation were not affected by the reform. This implies co-payments unlikely are the most important reason behind them, especially in the presence of a co-payment ceiling mechanism. Stratified differences in, for example, risk factors or access to primary care may have more impact. The gradual adaptation of newer non-insulin antidiabetic medicines during the study period is less likely to affect, as the available repertoire of antidiabetics in treatment and control groups was the same for all individuals in the group. However, before the reform, the increased co-payments were expected to especially affect people using newer and more expensive antidiabetics (Kurko et al., 2018). After the reform, a survey study found economic impacts being reported especially among people using the newer medicines (Suviranta, Timonen, Martikainen, & Aarnio, 2019). Thus, it remains possible that the socioeconomic impact of the reform is reflected in the consumption of newer non-insulin medicines. Analysis of this is beyond the scope of the current study design and should be evaluated in the future.

Previous studies have suggested an increase in economic difficulties (Aaltonen, Niemelä, & Prix, 2022; Rättö & Aaltonen, 2021; Lavikainen, Aarnio, Jalkanen, et al., 2020), negative developments in satisfaction of care (Lavikainen, Aarnio, Niskanen, Mäntyselkä, & Martikainen, 2020), and impacts related to the consumption of type 2 antidiabetics (Lavikainen, Aarnio, Jalkanen, et al., 2020; Lavikainen, Aarnio, Niskanen, et al., 2020) among individuals with type 2 diabetes after the reform. Regarding insulin consumption, a survey study found responders reporting increased insulin use due to cost (Lavikainen, Aarnio, Niskanen, et al., 2020). However, the population-level register study did not find an increase in the average consumption – instead, a decreasing trend was detected (Rättö, Kurko, Martikainen, & Aaltonen, 2021). The decreasing risk of insulin initiation in later years aligns with the findings of the register study. However, the results do not rule out the findings of the survey. Nevertheless, impacts on insulin initiation do not seem to vary systematically according to income level.

### 4.1 Strengths and limitations

Strengths of the current study include individual-level nationwide register data on all reimbursed medicine purchases used to define treatment and control groups and to provide information on insulin initiation. Comprehensive individual-level register data was also used to describe the background characteristics and socioeconomic status of the individuals. Finally, the quasi-experimental setting rising from the introduction of the reform allows for considerations of causality.

However, some caveats should be kept in mind: First, income directly reflects to an individual's ability to pay for health care services and it has often been used as an indicator of socioeconomic status when studying the inequalities in the use of health care services (e.g. van Doorslaer et al., 2000, 2006). However, in addition to income, socioeconomic position can be reflected in the educational or occupational status that were beyond the scope of the current study. Second, information on income is based on person-level and not on household-level taxable income. Thus, the sharing of the economic burden of medicines between the household members could not be accounted for. Taxable income also excludes some aspects of the Finnish social security system, such as social assistance, that could have mitigated the impact of increasing medicine costs for those eligible. Finally, the current study could not account for individual characteristics, such as weight or glycemic control, that would affect the clinical choice of diabetes medication. However, it is unlikely that these characteristics, on average, would differ greatly between treatment and control groups, as the groups were drawn from time points relatively close to each other.

### 4.2 Implications for policy and practice

Results of the study imply persisting differences between income quintiles in insulin initiation that should be taken into consideration in the future. However, despite the concerns about increased use of insulin for economic rather than clinical reasons, the differences were not affected by the reform. As health care systems can increase or reduce socioeconomic inequalities in health, changes in the system should also be carefully studied in the future to detect potential negative impacts on equality.

### 4.3 Further research

The impact of the reform on the newer non-insulin antidiabetics should be studied in the future. More analysis on the reasons behind socioeconomic differences in insulin initiation is also needed.

## 5 Conclusions

This study showed that the risk of insulin initiation was lower in the later years of the study period (2011–2019), which is in line with developing treatment practices of type 2 diabetes. Additionally, individuals in lower socioeconomic positions had a higher risk of initiation. However, no inequalities on how the reform affected the risk of insulin initiation were found between income quintiles, implying co-payments unlikely are the most influential factor behind persisting inequalities in insulin initiation among individuals with type 2 diabetes in Finland.

## Ethics statement

As the study was based only on administrative, secondary register data, no Ethics Board approval was required under Finnish law. The data used in the study were fully pseudonymized before accessing, and all data preparation and linkage in the study were done with pseudo-identifiers. As the register holder, the Social Insurance Institution of Finland (Kela) approved the use of the data for the current study.

## Funding

This research did not receive any specific grant from funding agencies in the public, commercial, or not-for-profit sectors.

## Author statement

HR was responsible for conceptualization, data curation, methodology, formal analysis, writing (original draft, review & editing) of the study.

## Declaration of competing interest

The author declares that they have no conflict of interest.

## Data Availability

The authors do not have permission to share data.
