# Supplementary material for: Socioeconomic inequalities in insulin initiation among individuals with type 2 diabetes – A quasi-experimental nationwide register study
Source: SSM Popul Health. 2022 Aug 9;19:101178. doi: 10.1016/j.ssmph.2022.101178 (PMC9399379; doi:10.1016/j.ssmph.2022.101178)

Supplemental table 1. Flow charts for treatment and control group formation. All data linkage is done with pseudo-identifiers. ATC=Anatomic Therapeutic Classification

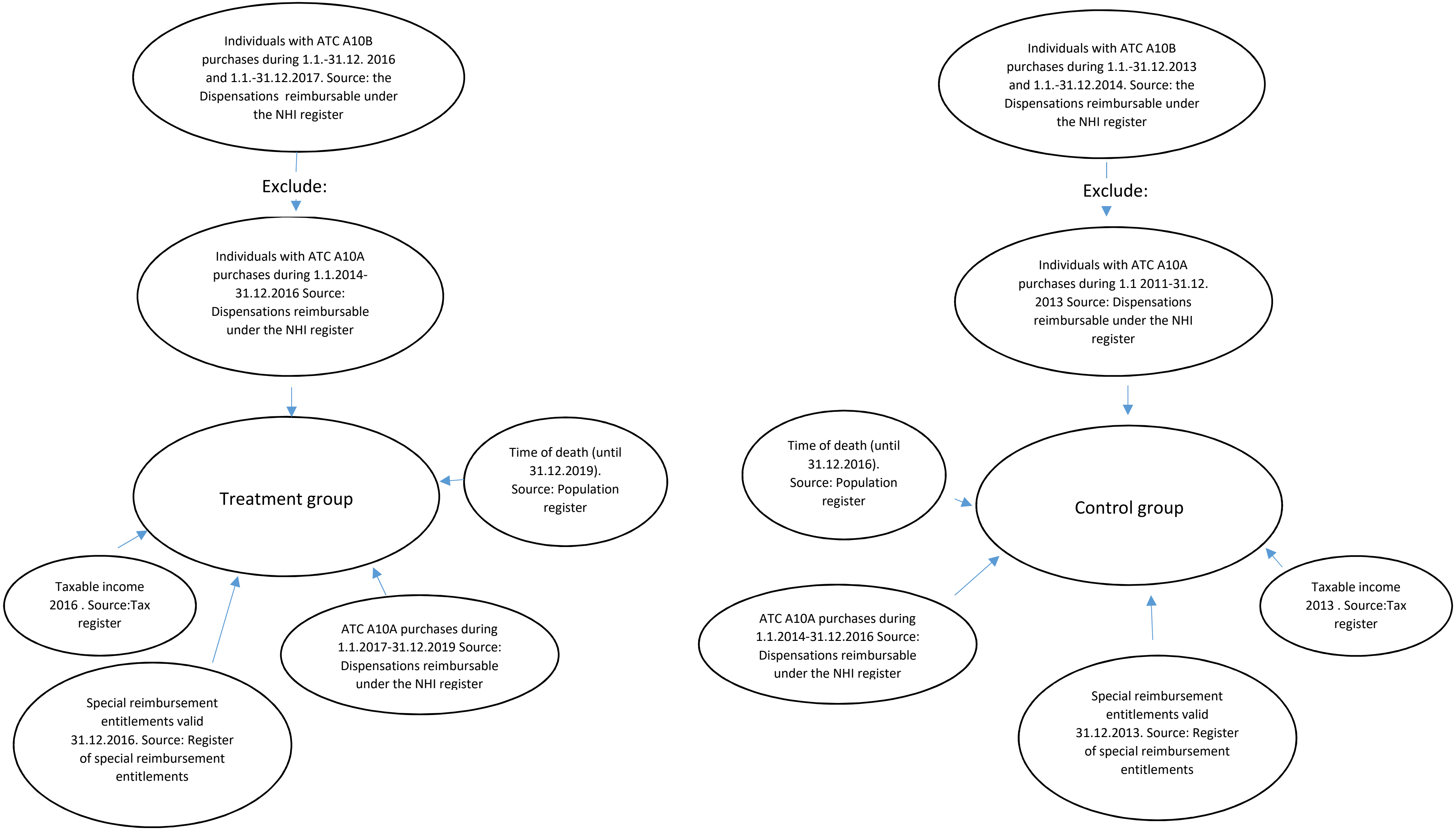

Supplement: Multimedia component 1 [file mmc1.pdf]
