# Supplementary material for: Socioeconomic inequalities in insulin initiation among individuals with type 2 diabetes – A quasi-experimental nationwide register study
Source: SSM Popul Health. 2022 Aug 9;19:101178. doi: 10.1016/j.ssmph.2022.101178 (PMC9399379; doi:10.1016/j.ssmph.2022.101178)
Supplement: Multimedia component 2 [file mmc2.docx]

Supplemental table 1.

**Individuals receiving reimbursements for different non-insulin antidiabetics 2011-2019, as share (%) of all individuals receiving reimbursements for non-insulin antidiabetics (A10B)**

|  | A10BA Biguanides (%) | A10BB Sulfonylureas (%) | A10BD Combinations of oral blood glucose lowering drugs (%) | A10BG Thiazolidinediones (%) | A10BH Dipeptidyl peptidase 4 (dpp-4) inhibitors (%) | A10BJ Glucagon-like peptide-1 (glp-1) analogues (%)* | A10BK Sodium-glucose co-transporter 2 (sglt2) inhibitors (%)* | A10BX Other blood glucose lowering drugs, excl. Insulins (%)* |
| --- | --- | --- | --- | --- | --- | --- | --- | --- |
| 2019 | 78,5 | 1,8 | 12,1 | 1,3 | 26,8 | 6,6 | 18,9 | 0,5 |
| 2018 | 78,7 | 2,3 | 12,2 | 1,4 | 28,0 | 5,1 | 15,8 | 0,5 |
| 2017 | 78,6 | 3,1 | 12,4 | 1,4 | 29,4 | 4,5 | 12,7 | 0,6 |
| 2016 | 79,0 | 4,1 | 12,6 | 2,0 | 31,0 | 4,9 | 8,2 | 0,7 |
| 2015 | 79,9 | 5,4 | 12,4 | 2,3 | 30,3 |  |  | 7,4 |
| 2014 | 81,0 | 6,8 | 12,2 | 2,5 | 28,3 |  |  | 5,6 |
| 2013 | 82,6 | 8,8 | 11,9 | 2,8 | 25,8 |  |  | 4,1 |
| 2012 | 84,4 | 11,9 | 11,3 | 3,4 | 22,6 |  |  | 3,4 |
| 2011 | 86,6 | 15,9 | 9,8 | 4,1 | 18,3 |  |  | 2,5 |

* Before 2016, GLP-1 analogues (first reimbursed in 2011) and SGLT2 inhibitors (first reimbursed in 2013) were classified in A10BX.

Source: Statistical database Kelasto, Kela. https://www.kela.fi/web/en/statistical-database-kelasto#Medicines
